# Constructing Gas Transmission Pathways in Two-Dimensional Composite Material ZIF-8@BNNS Mixed-Matrix Membranes to Enhance CO_2_/N_2_ Separation Performance

**DOI:** 10.3390/membranes13040444

**Published:** 2023-04-19

**Authors:** Fei Guo, Wu Xiao, Canghai Ma, Xuehua Ruan, Gaohong He, Hanli Wang, Zhendong Yang, Xiaobin Jiang

**Affiliations:** 1 State Key Laboratory of Fine Chemicals, Department of Chemical Engineering, Dalian University of Technology, 2 Linggong Road, Dalian 116024, China; 2 Shandong Huaxia Shenzhou New Material Co., Ltd., Zibo 256401, China

**Keywords:** BNNS, composite material, mixed matrix membranes, transmission pathway, gas separation

## Abstract

Two-dimensional (2D) nanomaterials, due to their high aspect ratio and high specific surface area, which provide a more tortuous pathway for larger gas molecules, are frequently used in membrane separation. However, in mixed-matrix membranes (MMMs), the high aspect ratio and high specific surface area of 2D fillers can increase transport resistance, thereby reducing the permeability of gas molecules. In this work, we combine boron nitride nanosheets (BNNS) with ZIF-8 nanoparticles to develop a novel material, ZIF-8@BNNS, to improve both CO_2_ permeability and CO_2_/N_2_ selectivity. Growth of ZIF-8 nanoparticles on the BNNS surface is achieved using an in-situ growth method where the amino groups of BNNS are complexed with Zn^2+^, creating gas transmission pathways that accelerate CO_2_ transmission. The 2D-BNNS material acts as a barrier in MMMs to improve CO_2_/N_2_ selectivity. The MMMs with a 20 wt.% ZIF-8@BNNS loading achieved a CO_2_ permeability of 106.5 Barrer and CO_2_/N_2_ selectivity of 83.2, surpassing the Robeson upper bound (2008) and demonstrating that MOF layers can efficiently reduce mass transfer resistance and enhance gas separation performance.

## 1. Introduction

The widespread use of cars as a primary means of transportation has contributed to increased global CO_2_ emissions, as the number of vehicles owned in many cities, including New York, Tokyo, and Beijing, has exceeded 5 million. Further, burning fossil fuels in natural gas- and coal-fired power plants also contributes significantly to global CO_2_ emissions. The concentration of CO_2_ in natural gas power plants varies from 4 to 6%, which is lower than in coal power plants. This has resulted in a pressing need to minimize the increase in CO_2_ concentrations in our environment, leading to extensive research into carbon capture and storage processes. In comparison with conventional gas separation processes, such as cryogenic distillation, absorption, and pressure swing adsorption, membrane separation processes have gained significant attention due to their numerous advantages, including low energy consumption, ease of scale-up, and greater energy efficiency, when compared to other gas separation methods [1,2]. Effective separation methods are crucial to decrease the CO_2_ concentration in the atmosphere, making membrane separation a promising technology for mitigating the negative effects of carbon emissions on the environment.

Commercial polymer membranes are commonly used because of their well-established synthesis [3,4,5], but these membranes cannot attain better separation performance in both permeability and selectivity [6]. The combination of organic and inorganic materials and polymeric membranes to form mixed matrix membranes (MMMs) [7,8] to break through the Robeson upper bound has been studied with the aim of combining the advantages of nanomaterials and polymers. In the MMMs, different types of nanomaterials were included in the polymer matrix.

Among the various possible fillers usually used, due to their large surface area, tunable pore size, and high porosity, metal–organic frameworks (MOFs) have attracted much attention and are extensively used in various fields [9,10,11,12,13]. One type of MOF is zeolite imidazole frameworks (ZIFs). They have an isomorphism to zeolites, and ZIF-8 is a well-known ZIF family member. Compared with CO_2_, ZIF-8 has a pore size of 3.4 Å [14,15,16]. Thus, ZIF-8 is a suitable choice as a filler to prepare MMMs for CO_2_ separation via a sieving mechanism [17,18]. However, according to the literature, the effective aperture of ZIF-8 is not rigid and much larger (4.0–4.2 Å) than its XRD-derived aperture of 3.4 Å, attributable to the rotation of 2-methylimidazole linkers [19,20]. In addition, an excellent CO_2_ permeability has been demonstrated by ZIF-8-based MMMs, but CO_2_/N_2_ selectivity decreased [21,22], which is attributed to the poor compatibility between micron-sized ZIF-8 and the polymer matrix causing interfacial voids and defects. In order to solve this problem, Abolfaz et al. [23] loaded ZIF-8 nanoparticles with ethylenediamine (ED) amine and incorporated that into a Pebax-1074 matrix to fill the interfacial voids and enhance the compatibility. Filler loadings were up to 30 wt.% in the MMMs after being modified, and the CH_4_ and CO_2_ permeability was up to 14.2 and 344 Barrer, respectively. The microemulsion-based mixed linker strategy was proposed by Ding et al. [24], who introduced amino groups into the mixture during ZIF-8 growth in order to enhance both interfacial compatibility and gas separation performance. Using MMM doped with 6 wt.% of NH_2_-ZIF-8(10), the CO_2_ permeability increased by 53.3% and 107.6% compared with a pure Pebax membrane and ZIF-8/Pebax MMM.

Two-dimensional (2D) nanostructured materials have been widely used in various fields, such as optoelectronics [25], functional materials, energy storage, and gas separation [26,27,28], because of their nanometer thickness with its high ratio, which minimizes the transport resistance.

Hexagonal boron nitride (h-BN), also known as ‘white graphene’ or ‘non-carbon graphene’, is composed of layers of boron and nitrogen atoms arranged in a hexagonal lattice structure, connected by strong covalent (sp^2^) bonds. Boron nitride nanosheets (BNNS) possess unique characteristics that complement those of graphene, such as a wide energy band gap, excellent electrical insulation, high thermal stability, and exceptional chemical inertness [29,30,31]. These characteristics make BNNS a highly promising material for a range of applications in electronic devices, composites, and other fields. Therefore, BNNS have been widely used in thermal conductive insulating material [32,33,34,35], waterproof coatings [36], hydrogen storage [37], and water purification [38,39]. However, the incorporation of BNNS into the polymer matrix will significantly improve the selectivity with a significant decrease in the permeability. For example, using amino-functionalized boron nitride nanosheets, Wang et al. [40] prepared FBN-XTR nanocomposite membranes incorporating a crosslinked and thermally rearranged polyimide at 425 °C (XTR). A 1 wt.% FBN membrane demonstrated a H_2_ permeability of 110 Barrers and a H_2_/CH_4_ selectivity of 275. In contrast, the heat-treated XTR membrane showed a H_2_/CH_4_ selectivity of 24.1 and H_2_ permeability of 210 Barrers. Ahmed et al. [41] used BNNS and PIM-1 to prepare MMMs to reduce the physical aging of PIM-1 and tested them in a CO_2_/CH_4_ (1:1, *v*:*v*) binary gas mixture system. The permeability test results showed that CO_2_ permeability decreased with increasing amounts of BNNS, up to the tested 2 wt.% loading, suggesting that the addition of BNNS increases the tortuosity for gas molecules, slowing down diffusion and perhaps partially blocking some PIM-1 pores. Moreover, after 414 days, the CO_2_ permeability of the MMMs decreased by 22%, while that of neat PIM-1 decreased by 58%. Both membranes exhibited a permeability decrease and a selectivity increase at low filler loadings. Therefore, the question of how to incorporate 2D materials into MMM without sacrificing its permeability is an important question.

In this work, we combine BNNS with ZIF-8 to construct a composite material to investigate the use of 2D materials in MMMs and their effect on the sacrifice of permeability. BNNS were selected as the template, and we used the in-situ growth method to grow ZIF-8 on their surface. ZIF-8 layers construct a CO_2_ transmission pathway to reduce transmission resistance and improve gas permeability. BNNS require barriers to block gas molecules and improve selectivity. Then, the composite materials are combined with a Pebax-1657 matrix to fabricate MMMs. We chose Pebax-1657, a commercial rubbery polymer, as the polymer matrix. It is composed of polyamide (PA) and polyether (PEO) blocks. PEO segments have a high CO_2_ permeability capability, and the PA segment has a strong mechanical strength [42,43]. Finally, the fundamental separation mechanism of ZIF-8@BNNS MMM is considered, along with an investigation into how the filler content and feed pressure impact the CO_2_ permeability and CO_2_/N_2_ selectivity.

## 2. Results and Discussion

### 2.1. FTIR and XRD

**Figure 1:**
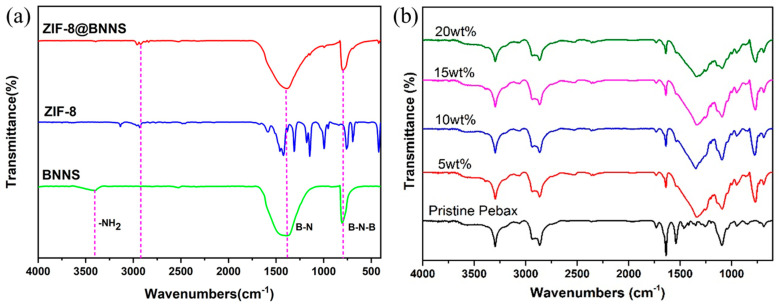
FTIR spectrum of (**a**) ZIF-8, BNNS, and ZIF-8@BNNS and (**b**) Pristine Pebax and ZIF-8@BNNS/Pebax MMMs with different loadings.

Figure 1a shows the FTIR spectra of the materials. The spectrum of BNNS exhibited a band at around 3400 cm^−1^ associated with—NH_2_, which indicates amino groups on the surface of the BNNS. Another broad band appeared at about 1400 cm^−1^ in the BNNS spectrum, corresponding to the B–N bonds. The B–N–B band appeared in 808 cm^−1^, corresponding to in-plane B–N transverse stretching vibration and out-of-plane B–N–B bending vibration. In the ZIF-8 spectrum, the absorption peaks at 2930 cm^−1^ were in response to C–H groups from ZIF-8. Both these bands were also observed in the spectrum of ZIF-8@BNNS but not in ZIF-8, confirming that BNNS and ZIF-8 were both present in the synthesized composite fillers.

FTIR analyses were performed on pristine Pebax and ZIF-8@BNNS/Pebax MMMs with different filler loadings, as shown in Figure 1b. The FTIR spectrum of ZIF-8@BNNS/Pebax MMMs showed new bands at about 1300 cm^−1^ associated with the B–N bonds and about 808 cm^−1^ associated with the B–N–B bonds, indicating the successful incorporation of BNNS into the MMM. No new bands appeared, nor were any old bands broken, indicating that ZIF-8@BNNS and Pebax-1657 formed a physical blend and achieved a perfect integration between the composite material and the MMM matrix.

**Figure 2:**
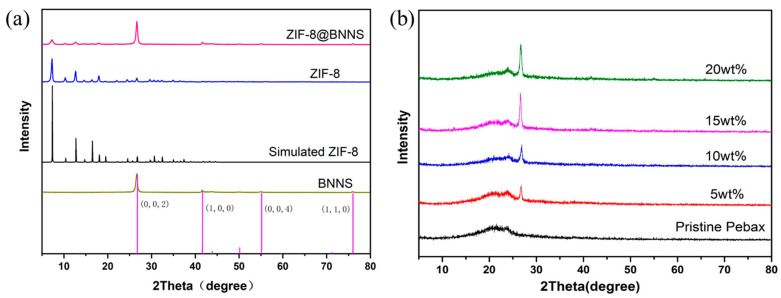
XRD patterns of (**a**) ZIF-8, BNNS, and ZIF-8@BNNS and (**b**) Pristine Pebax and MMMs with different loadings.

The X-ray diffraction (XRD) patterns of the fillers are displayed in Figure 2. Figure 2a indicates that BNNS displayed a diffraction peak at 2θ = 26.72° and 55. 07°, corresponding to the 002 and 100 peaks. In the composites of ZIF-8@BNNS, the characteristic diffraction peaks of BNNS were simultaneously observed. Moreover, it is obvious that all the diffraction peaks of ZIF-8 and ZIF-8@BNNS were in good agreement with the simulation of ZIF-8, revealing that a typical ZIF-8 sodalite structure had successfully grown on the BNNS’s surface. Compared with the ZIF-8 spectrum, all diffraction peaks in the ZIF-8@BNNS were attenuated to some extent because ZIF-8 particles under constrained growth on the BNNS surface would be a little more imperfect than the particles synthesized in free space. The diffraction peak positions revealed that the ZIF-8@BNNS composites were well-matched with the neat ZIF-8 fillers. Moreover, the incorporation of ZIF-8 did not disrupt the crystal structure.

The XRD patterns of MMMs provide insights into the crystal structures of the polymer matrix and filler. As depicted in Figure 2b, all MMMs exhibited two distinct diffraction peaks at approximately 2θ = 20.0° and 24.0°, which are characteristic peaks of the poly(ethylene oxide) (PEO) and polyamide (PA) phases in the Pebax polymer matrix. Compared with the pristine membrane, the diffraction peaks at approximately 2θ = 26.6° in the XRD patterns of MMMs confirmed the successful dispersion of ZIF-8@BNNS within the Pebax matrix. This indicates that the filler was effectively dispersed within the matrix without any alteration of the crystal structure of the fillers.

### 2.2. Materials and MMMs Characterization

#### 2.2.1. ZIF-8@BNNS and MMMs Morphology

**Figure 3:**
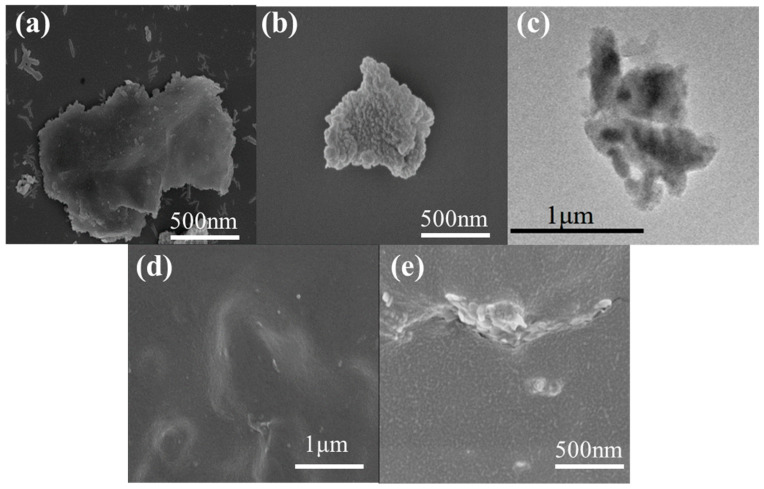
SEM and TEM pictures of (**a**) BNNS, (**b**,**c**) ZIF-8@BNNS composite materials, and (**d**,**e**) the surface and cross-section of the ZIF-8@BNNS/Pebax MMM loaded with 20 wt.%.

The ZIF-8@BNNS and MMMs’s morphology was characterized using SEM and TEM. As shown in Figure 3, the pure BNNS exhibited a smooth surface. However, as shown in Figure 3b, the ZIF-8@BNNS’s surface was rough, and a continuous ZIF-8 layer was coated on its surface, indicating the successful preparation of the ZIF-8@BNNS composite material. The TEM image of the ZIF-8@BNNS composite material in Figure 3c revealed the composite structure. The HRTEM and SAED images of ZIF-8@BNNS are also provided in Figure S4. It can be seen from the figure that the in-situ growth of ZIF-8 on the surface of BNNS was caused by the 002 plane of BNNS. Furthermore, the SEM image and energy-dispersive X-ray spectroscopy (EDS) mapping of Zn and B elements of doped BNNS are presented in Figure S1, which confirmed the successful growth of ZIF-8 on the surface of BNNS.

The SEM images presented in Figure 3d,e, Figures S2 and S3 provide a detailed view of the membrane surface and cross-section of both the pristine membrane and the MMMs. The homogenous distribution of the filler is clearly visible on the membrane surface, while the cross-section image indicated there were no obvious interfacial defects between the composite material and the matrix. This phenomenon can be attributed to the effective filler dispersion achieved by the ZIF-8 layer, which prevents agglomeration. Moreover, the presence of BNNS in the MMMs minimizes the formation of large ZIF-8 agglomerates. EDS mapping of MMM loaded with 20 wt.% is shown in Figure S5. We can observe from the figure that Zn, N, and B elements were uniformly distributed on the membrane surface, which confirmed the homogenous distribution of the filler in MMMs. These results demonstrate the promising potential of the composite membrane in practical applications.

#### 2.2.2. TGA Analysis

**Figure 4:**
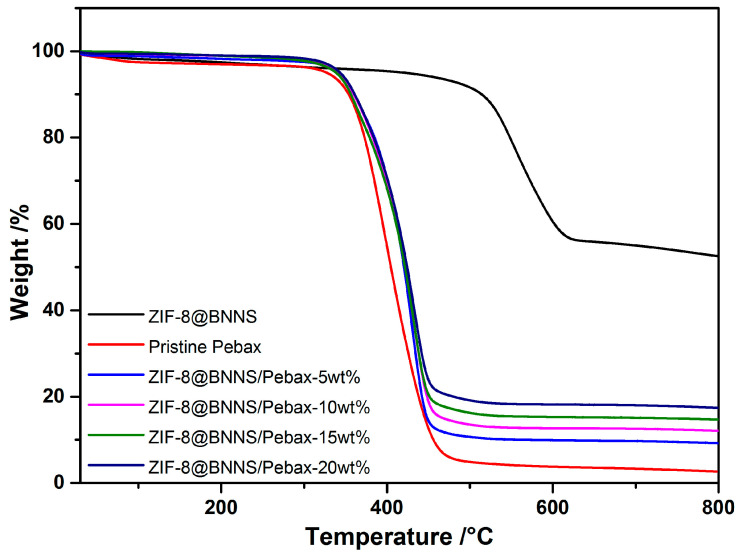
TGA curves of ZIF-8@BNNS and ZIF-8@BNNS/Pebax membranes.

Thermal stability is a crucial factor in determining the suitability of the composite material for various applications, which can be evaluated using TGA. As shown in Figure 4, the weight loss of ZIF-8@BNNS can be divided into three stages. The first stage, in the range of 30 to 510 °C, corresponds to the evaporation of the residual or adsorbed solvent, resulting in a slight mass loss. The second stage, from 510 °C to 600 °C, is primarily attributed to the decomposition of the ZIF-8 framework structure grown on the surface of BNNS. At temperatures exceeding 600 °C, only the non-decomposed ZnO and BNNS remained because of the stable chemical properties of BNNS. These results demonstrate the high thermal stability of the composite material and indicate its potential for use in various high-temperature applications.

The thermal stability of the membranes was evaluated by analyzing their weight loss, and the results are presented in Figure 4. All the membranes exhibited similar decomposition trends, characterized by three weight-loss stages. In the first stage, occurring in the temperature range of 25 to 350 °C, the weight loss can be attributed to the evaporation of water and organic solvents from the membrane. The second stage, observed between 350 and 450 °C, is primarily attributed to the carbonization and pyrolysis of the Pebax polymer backbone, which is consistent with previous findings reported by Dai et al. [44]. Beyond 450 °C, the weight loss became less significant. These results demonstrate the good thermal stability of the membranes, which is a crucial factor for their successful application in high-temperature environments.

#### 2.2.3. DSC Analysis

The thermal properties of both the neat Pebax and MMMs were assessed using DSC analysis. The detailed crystallinity information of the MMMs is presented in Table 1. The DSC analysis revealed two characteristic melting temperatures (*T*_m_) at 14.74 °C and 203.82 °C, corresponding to the PEO and PA segments in the Pebax membrane, respectively. Furthermore, the incorporation of ZIF-8@BNNS composite materials into the polymeric matrix led to a slight increase in the *T*_g_ values. Interestingly, the polymer matrix did not significantly affect the incorporation between the composite materials and the thermal properties of the prepared membranes. These results provide valuable insights into the thermal behavior of the MMMs and their potential applications in various fields.

### 2.3. BET Characterization of the Material

**Figure 5:**
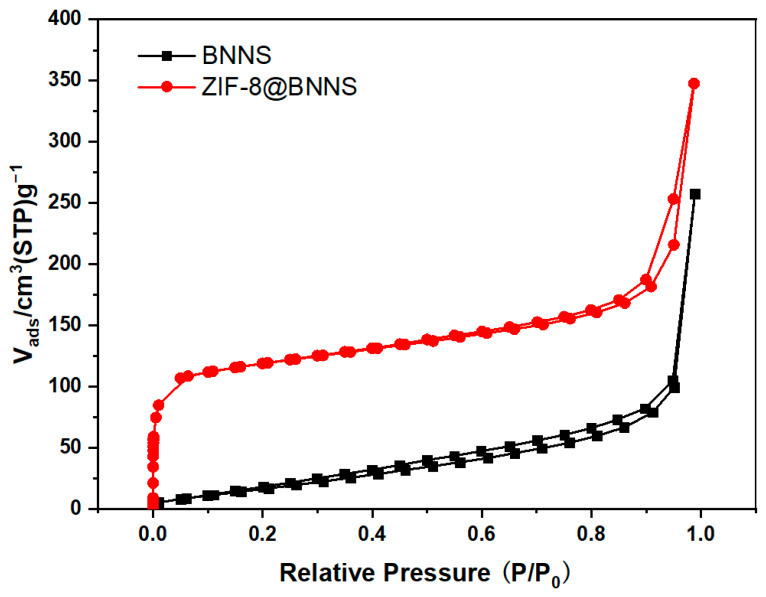
Nitrogen adsorption–desorption curve of ZIF-8@BNNS composite materials.

The BET surface area, BJH pore volume, and average pore size of the BNNS and ZIF-8@BNNS composite material were measured using N_2_ adsorption isotherms. As shown in Figure 5 and Table 2, both samples exhibited type IV adsorption isotherms with characteristic hysteresis loops, indicating the presence of numerous mesoporous structures. The in-situ growth of ZIF-8 on the BNNS surface increased the number of pores, resulting in a significantly higher BET surface area of 448.36 m^2^ g^−1^ compared to the value of 76.35 m^2^ g^−1^ for BNNS alone. This enhancement can be attributed to the porous channels provided by the ZIF-8 layer, which facilitates the transportation of gas molecules through the porous layer. The average pore size of ZIF-8-BNNS creates small pores and reduces the average pore size. These results demonstrate the potential of ZIF-8@BNNS composite materials as highly efficient adsorbents and catalysts for various applications.

### 2.4. Gas Permeation Measurements

**Figure 6:**
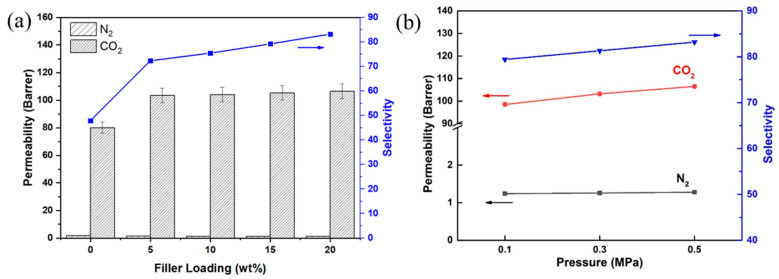
Gas permeation tests of ZIF-8@BNNS/Pebax MMMs (**a**) CO_2_ and N_2_ permeability and CO_2_/N_2_ selectivity of the membranes with different ZIF-8@BNNS loadings (0, 5 wt.%, 10 wt.%, 15 wt.%, and 20 wt.%), (**b**) CO_2_ permeability and CO_2_/N_2_ selectivity at different pressures (20 wt.% loading).

To investigate the effect of the ZIF-8@BNNS filler content on the permeability of MMMs, pure gas permeation tests were conducted on the MMMs with N_2_ and CO_2_ at 3 bar and room temperature (25 °C). The gas permeability and selectivity of the MMMs with different filler contents, ranging from 0 to 20 wt.%, were measured, as shown in Figure 6a. Each membrane was tested three times, and the average values calculated. The results indicated that the CO_2_ permeability gradually increased with increased filler loading, resulting in an increment of 130% compared to pristine Pebax when the ZIF-8@BNNS loading was 20 wt.%. With a 20% filler loading, the ZIF-8@BNNS/Pebax MMM exhibited an increase in permeability from 80.97 to 106.5 Barrers due to ZIF-8′s high porosity and flexible framework, which improved CO_2_ diffusion. Moreover, the N_2_ permeability slightly decreased from 1.67 to 1.28 Barrers. This phenomenon can be attributed to the microstructure of ZIF-8@BNNS. The large ZIF-8 cavity provides high gas diffusivity and introduces a larger free volume, which accelerates the diffusion of CO_2_. Further, BNNS acted as a barrier to block gas molecules in the membranes, leading to a decrease in N_2_ permeability. Furthermore, the ZIF-8@BNNS/Pebax MMM demonstrated higher CO_2_/N_2_ selectivity compared to the pristine membrane due to the ZIF-8 layer constructing a continuous pathway for CO_2_ transmission. Consequently, the significant increase in CO_2_ permeability resulted in a better separation performance than the pristine membrane.

Detailed analysis of the gas diffusivity and solubility coefficients of ZIF-8@BNNS/Pebax membranes are summarized in Table 3. When the filler loading was increased from 0 to 20 wt.%, the diffusivity coefficients of CO_2_ increased, indicating that the increase in CO_2_ permeability was mainly due to the increment in the diffusivity coefficient of CO_2_. The addition of fillers increases the free volume in the membrane, and the free volume increases as the filler loading increases. The existence of BNNS creates a tortuous pathway for gas transport that leads to decreased gas permeability, especially for larger gas molecules, so the diffusivity coefficient of N_2_ decreases as the filler loading increases. The introduction of fillers results in a disturbed polymer chain packing, and an increased interfacial volume can be created, thereby increasing gas diffusivity via introducing more alternative routes.

The prepared membranes were subjected to permeation tests at different pressures of up to 0.5 MPa, and the results are presented in Figure 6b. Generally, the separation performance of rubbery polymeric membranes is influenced by both the solubility and diffusion rate of the gas, which increase with the feed pressure. Accordingly, the CO_2_ permeability of the membranes increased with increasing pressure, from 0.1 MPa to 0.5 MPa. Inclusion of the ZIF-8@BNNS composite material into the membranes further improved their separation performance with increasing pressure, resulting in a significant enhancement of gas separation.

**Figure 7:**
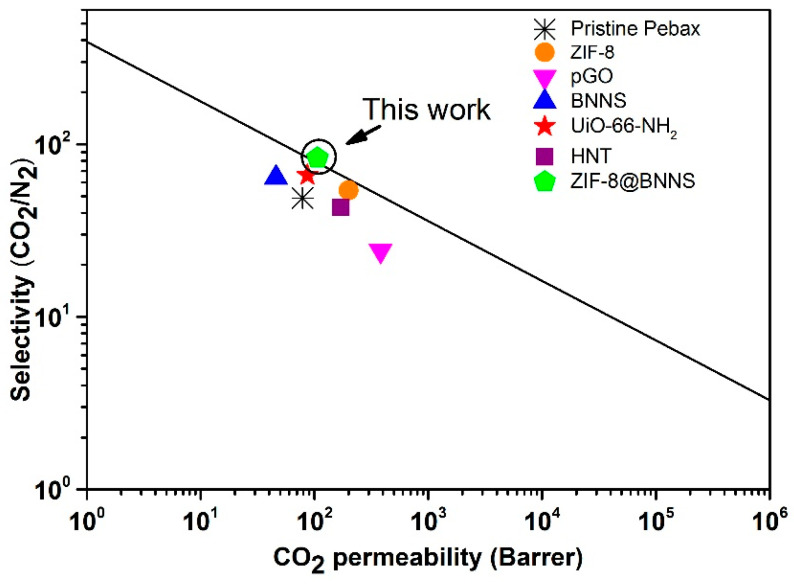
The relationship between the CO_2_ permeability and the CO_2_/N_2_ selectivity of the MMMs prepared in this work and the literature.

Various types of fillers have been used to improve the separation performance of CO_2_/N_2_ by Pebax membranes, such as ZIF-8, pGO, BNNS (Detailed data in Table S1), UiO-66-NH_2_, and halloysite nanotube (HNT), and their reported data are summarized in Table 4. In addition, we compared our results with the Robeson upper bound (2008) [6]. Among all the fillers, the 20 wt.% ZIF-8@BNNS/Pebax MMM exhibited the best CO_2_/N_2_ separation performance, surpassing the Robeson upper bound (2008), as illustrated in Figure 7. The remarkable enhancement in CO_2_/N_2_ separation performance of the ZIF-8@BNNS/Pebax membrane indicated that it performs as well as or better than most of the other reported membranes. The incorporation of ZIF-8@BNNS improved the interface morphology and gas separation performance and is a promising candidate for CO_2_ capture and storage.

**Figure 8:**
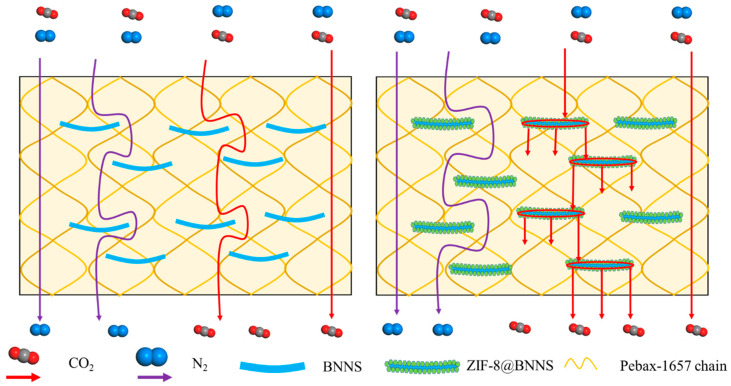
Comparison in mass transfer pathways for MMMs containing BNNS and ZIF-8@BNNS.

The mechanism of ZIF-8@BNNS is illustrated in Figure 8. A new type of material is created by combining the MOF material ZIF-8 with 2D boron nitride nanosheets. The BNNS in the MMMs act as a barrier to block gas transmission pathways, forcing gas molecules to pass via interfacial diffusion and providing a more tortuous pathway for larger gas molecules. In contrast, the ZIF-8 layer surrounding BNNS acts as an expressway, significantly reducing mass transfer resistance compared to the BNNS barriers alone. There will still be a small number of N_2_ molecules passing through the ZIF-8 layer attributable to the rotation of 2-methylimidazole linkers. As a result, the ZIF-8@BNNS/Pebax MMMs exhibited a remarkable boost in gas separation performance. This combination of materials offers a promising approach to improving gas separation processes.

## 3. Methods and Materials

### 3.1. Materials

The synthesis of ZIF-8@BNNS involved the use of 2-methylimidazole (2 mim) and zinc nitrate hexahydrate (Zn(NO_3_)_2_⋅6H_2_O, AR), which were purchased from GUOYAO (Shanghai, China). Boron nitride nanosheets (AR) were obtained from Nanjing XFNANO (Nanjing, China). Ethanol (ETOH, AR) and methanol (MEOH, AR) used in the synthesis process were sourced from Tianjin Damao (Tianjin, China). High-purity N_2_ and CO_2_ gases for gas permeation tests were supplied by the Dalian Institute of Chemistry and Physics (Dalian, China). The membrane matrix and pristine membranes were made using Pebax-1657 (AR), which was obtained from Arkema (Colombes, France). All other reagents used in the process were purchased from commercial sources and used without further treatment.

### 3.2. Preparation of ZIF-8@BNNS Composite Fillers

To synthesize ZIF-8@BNNS, 0.4 g of BNNS was dispersed in 100 mL of MEOH, and 4 g of Zn(NO_3_)_2_⋅6H_2_O was added to the mixture. The resulting mixture was sonicated for 30 min to allow chelation of Zn^2+^ ions with the—NH_2_ groups on the BNNS surface. The Zn^2+^-doped BNNS suspension was then replaced with fresh methanol to create an environment suitable for ZIF-8 synthesis. Next, 11.03 g of 2-methylimidazole was dissolved in 100 mL of MEOH and added to the suspension containing the Zn^2+^-doped BNNS. The resulting mixture was sonicated and stirred continuously for 6 h at room temperature (25 °C) to synthesize ZIF-8 particles on the surface of BNNS. The resulting filler samples were refined to remove any remaining reactants by sequential centrifugal separation (at 10,000 r/min) and washed with water or methanol three times. Finally, the refined fillers were dried at 60 °C under vacuum for 12 h to obtain powder samples for further use.

In order to prove the existence of ZIF-8 in ZIF-8@BNNS and for further use, we synthesized ZIF-8 particles separately. ZIF-8 nanocrystals were synthesized in a typical synthesis, 4.41 g of Zinc nitrate hexahydrate and 4.87 g of 2 methylimidazole were dissolved separately in 300 mL methanol (designated as solution A and solution B, respectively). Then, solution B was added to solution A rapidly under vigorous stirring with a final molar ratio of 1:4:1000 (Zn:Hmim:methanol). After a 1-h reaction, the turbid solution was centrifuged and washed with methanol three times. A quantity of the freshly synthesized nanoparticles was re-dispersed in methanol for further characterization.

### 3.3. Membrane Preparation

To prepare the ZIF-8@BNNS/Pebax MMM, a specific amount of ZIF-8@BNNS powders was ground and dispersed into a mixed solvent of ethanol and water with a mass ratio of 7:3, followed by 5 min of ultrasonic treatment. Pebax-1657 particles were then added to the mixture, and the resulting solution was heated under stirring and refluxing at 80 °C for 4 h, yielding a membrane solution containing a 3.0 wt.% Pebax polymer matrix. The solution was ultrasonically treated for an additional 2 h after heating to remove any remaining bubbles. The solution was then poured into flat PTFE molds at room temperature (25 °C) and left to dry for 12 h. Finally, the resulting mixture was dried in a vacuum oven at 40 °C for 24 h to evaporate the solvent. A similar procedure was followed to prepare the pristine membrane.

The ZIF-8@BNNS loading is defined as follows:

$$\mathrm{ZIF}\text{-}8@\mathrm{BNNS}\;\mathrm{loading}\;(\mathrm{wt}.\%)=\frac{m_{\mathrm{ZIF}\text{-}8@\mathrm{BNNS}}}{m_{\mathrm{ZIF}\text{-}8@\mathrm{BNNS}}+m_{\mathrm{Pebax}}}\times 100\%$$

where $m_{\mathrm{ZIF}\text{-}8@\mathrm{BNNS}}$ and $m_{\mathrm{Pebax}}$ are the mass of ZIF-8@BNNS and Pebax.

**Figure 9:**
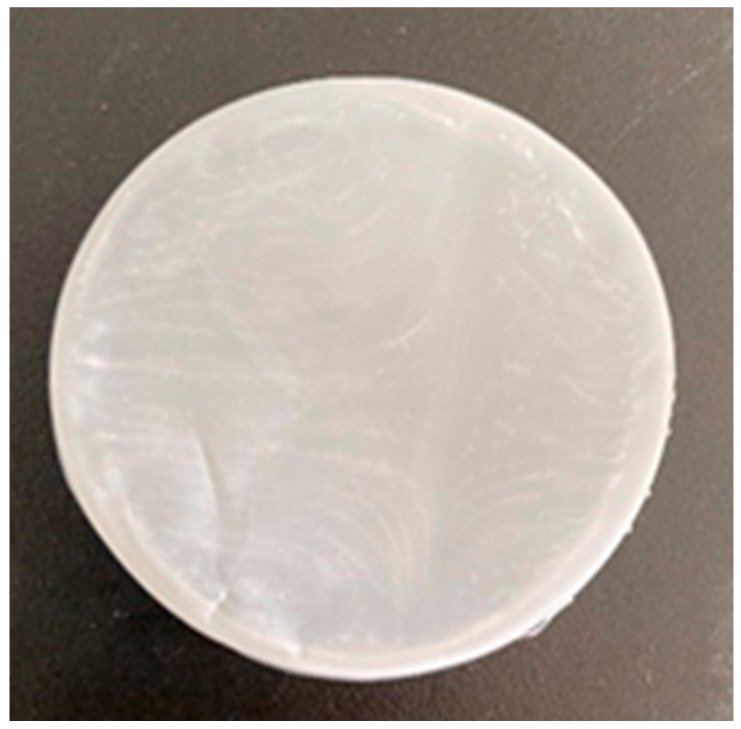
Image of a ZIF-8@BNNS/Pebax mixed matrix membrane.

ZIF-8@BNNS/Pebax MMMs with different loadings of 5, 10, 15, and 20 wt.% and a pure Pebax membrane were prepared. The membrane picture is shown in Figure 9. The thickness of each was about 75 to 90 μm.

### 3.4. Fillers and Membranes Characterization

Various tests were conducted to characterize both the materials and membranes. The morphology of the composite material ZIF-8@BNNS and the filler dispersion in the MMMs were examined using the Nova Nano SEM 450 scanning electron microscope at 20 kV. The sample for the composite material was prepared by dispersing and sonicating it in ethanol for 20 min, while cross-sections were prepared under liquid nitrogen. Rigaku SmartLab 9 kw X-ray diffraction was employed for wide-angle XRD analysis, and the diffraction angle 2θ was scanned between 5° and 80°, at a rate of 10°. Fourier transform infrared (FTIR) spectra were recorded using a MAGNA-560 spectrometer from the Bruker Company (Bremen, German) for wavenumbers ranging from 4000 to 400 cm^−1^. To measure the N_2_ adsorption–desorption isotherms of ZIF-8@BNNS, a porosity analyzer (ASAP2460) was used at 77 K. Additionally, thermogravimetric analyses (TGA) were performed using a Mettler Toledo TGA system. The thermal stability of ZIF-8@BNNS and MMMs were determined by thermogravimetric analysis (TGA) using a Mettler Toledo TGA/SDT 851e. The tests were performed at a heating rate of 10 °C min^−1^ under N_2_ flow from room temperature up to 800 °C. Samples weighing 3–5 mg were heated and kept at 100 °C for 12 h to remove the absorbed water and solvents. 

### 3.5. Permeability Experiment

The gas permeability experiment of MMMs was tested by a constant volume gas permeation device [48]. After degassing for at least 8 h, the gas permeability coefficient was tested by the constant volume method at 25 °C. The gas permeability calculation formula is as follows:

$$P=\frac{VL}{ART\left(p_{1}-p_{2}\right)}(\frac{dp_{2}}{dt})$$

The CO_2_/N_2_ selectivity ($\alpha_{CO_{2}/N_{2}}$) can be obtained as follows:

$$\alpha_{CO_{2}/N_{2}}=\frac{P_{CO_{2}}}{P_{N_{2}}}$$

The diffusivity (*D*) and solubility coefficients (*S*) were obtained from the literature, with calculation formulas as follows [48,49]:

$$D=\frac{L^{2}}{6\theta }$$

$$S=\frac{P}{D}$$

## 4. Conclusions

In this work, we focused on the development of Pebax membranes with enhanced CO_2_/N_2_ separation performance through the incorporation of a ZIF-8@BNNS composite material synthesized via in-situ growth. Various measurements were conducted to characterize the MMM’s structure and morphology, including FTIR, XRD, SEM, TGA, DSC, and BET. The results demonstrated excellent compatibility between the ZIF-8@BNNS matrix and Pebax. The incorporation of ZIF-8@BNNS into the Pebax membrane improved the CO_2_/N_2_ separation performance, resulting in a better CO_2_ permeability of 106.5 Barrers and CO_2_/N_2_ selectivity of up to 83.2 under a 20 wt.% ZIF-8@BNNS loading. The as-prepared MMMs showed simultaneous increases in CO_2_ permeability and CO_2_/N_2_ selectivity compared to pure Pebax membranes. The strategy of combining MOF with 2D materials has the potential to significantly enhance CO_2_ separation performance and overcome the limitations of two-dimensional materials.

## Figures and Tables

**Table 1:** Thermal properties of ZIF-8@BNNS/Pebax mixed matrix membranes.

Membranes	*T*_g_ (°C)	PEO	PA
		*T*_g_ (°C)	*T*_m_ (°C)
Pristine Pebax	−54.3	14.74	203.82
5 wt.%	−52.7	14.16	203.75
10 wt.%	−52.2	14.39	203.91
15 wt.%	−52.4	14.53	203.46
20 wt.%	−53.0	14.41	204.02

**Table 2:** BET data of BNNS and ZIF-8@BNNS.

Sample	BNNS	ZIF-8@BNNS
BET surface/m^2^∙g^−1^	76.35	448.36
Pore volume/cm^3^∙g^−1^	0.39	0.54
Average pore size/nm	3.41	1.08

**Table 3:** Gas diffusivity coefficients and solubility coefficients of ZIF-8@BNNS/Pebax membranes.

ZIF-8@BNNS Loading (wt.%)	*D*(CO_2_) ^a^	*S*(CO_2_) ^b^	*D*(N_2_) ^a^	*S*(N_2_) ^b^
0	10.43 ± 0.02	7.76 ± 0.03	8.25 ± 0.04	0.20 ± 0.01
5	12.59 ± 0.03	8.21 ± 0.04	5.96 ± 0.03	0.24 ± 0.02
10	12.66 ± 0.01	8.22 ± 0.02	5.8 ± 0.03	0.24 ± 0.01
15	12.79 ± 0.04	8.23 ± 0.02	5.77 ± 0.04	0.23 ± 0.02
20	12.94 ± 0.03	8.23 ± 0.04	5.6 ± 0.03	0.23 ± 0.01

^a^ Diffusivity coefficient (cm^2^/s) × 10^6^, ^b^ Solubility coefficient (cm^3^(STP)/cm^3^ cm Hg) × 10^4^.

**Table 4:** Comparison of CO_2_ separation performance of MMMs in this work and the literature.

Materials	Conditions	P_CO_2__ (Barrer)	α_CO_2_/N_2__	Ref.
Pebax-1657	5 bar, 25 °C	78.6	48.7	This work
ZIF-8/Pebax-1657	-	199.57	53.88	[45]
pGO/Pebax-2533	1 bar, 35 °C	380.44	24.19	[46]
BNNS/Pebax-1657	5 bar, 25 °C	45.96	64.01	This work
UiO-66@HNT/Pebax-1657	5 bar, 25 °C	119.08	76.26	[47]
ZCN/Pebax-1657	2 bar, 25 °C	110.5	84.4	[28]
ZIF-8@BNNS/Pebax-1657	5 bar, 25 °C	106.5	83.2	This work

## Supplementary Materials

The following supporting information can be downloaded at https://www.mdpi.com/article/10.3390/membranes13040444/s1.
