# Supplementary material for: Constructing Gas Transmission Pathways in Two-Dimensional Composite Material ZIF-8@BNNS Mixed-Matrix Membranes to Enhance CO2/N2 Separation Performance
Source: Membranes (Basel). 2023 Apr 19;13(4):444. doi: 10.3390/membranes13040444 (PMC10143403; doi:10.3390/membranes13040444)
Supplement: Supplementary file 1 [file membranes-13-00444-s001.zip › membranes-2286369-supplementary.pdf]

Supporting Information

Constructing gas transmission pathways of two-dimensional composite material ZIF-8@BNNS mixed matrix membranes to enhance CO_2/N_2 separation performance

Legend of Figures

Figure S1. SEM image and EDS mapping (Zn and B) of ZIF-8@BNNS.

Figure S2. SEM surface image of the ZIF-8@BNNS/Pebax membrane loaded with (a) 5 wt% (b) 10 wt% (c) 15 wt%

Figure S3. SEM cross-section image of the ZIF-8@BNNS/Pebax membrane loaded with (a) 5 wt% (b) 10 wt% (c) 15 wt% (d) 20 wt%

Figure S4. HRTEM images (a) of the ZIF-8@BNNS corresponding SAED pattern (b)

Figure S5. SEM image and EDS mapping (Zn and B) of MMM loaded with 20 wt%

Legend of Tables

Table S1. CO_2 permeability and CO_2/N_2 selectivity of MMMs with different BNNS loadings and different pressures

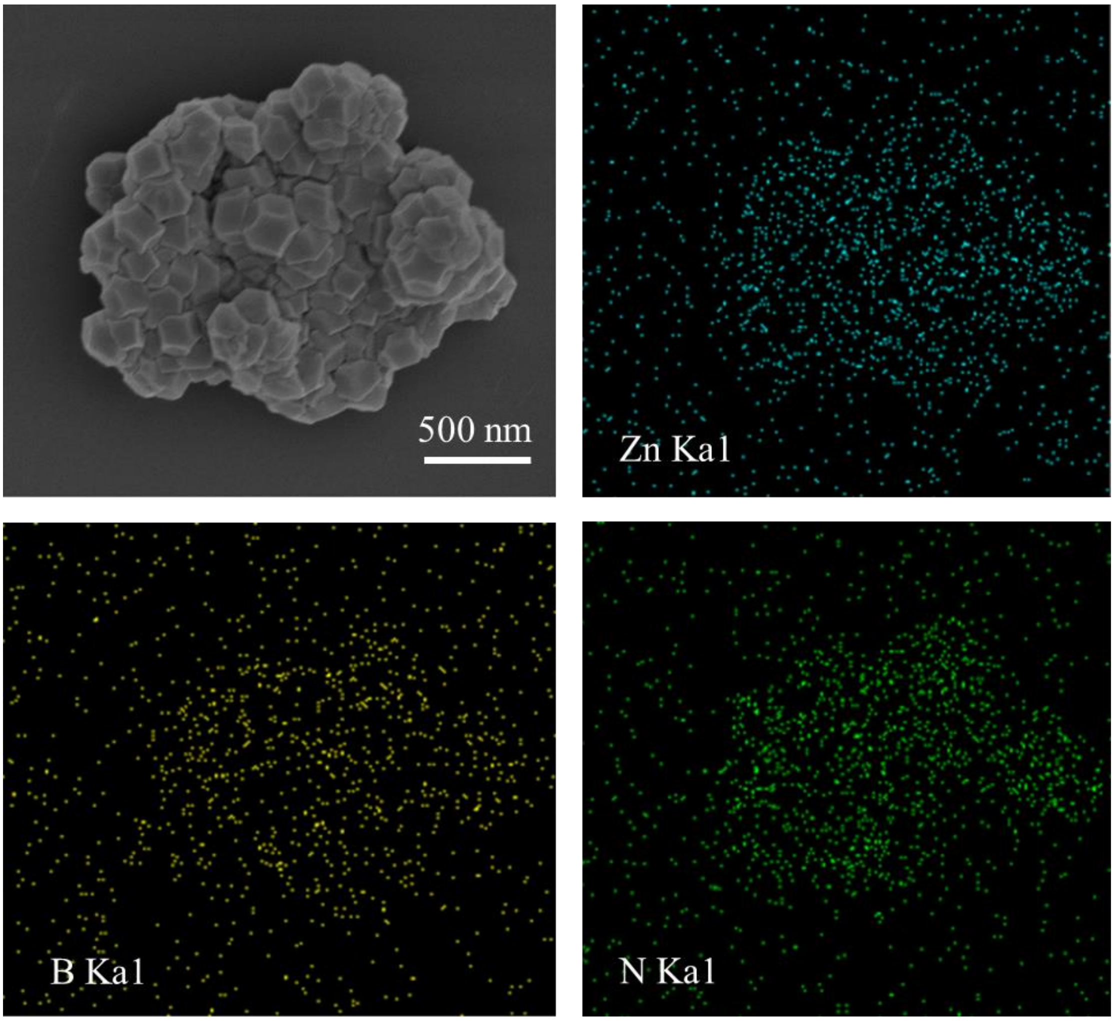

Figure S1. SEM image and EDS mapping (Zn, B and N) of ZIF-8@BNNS.

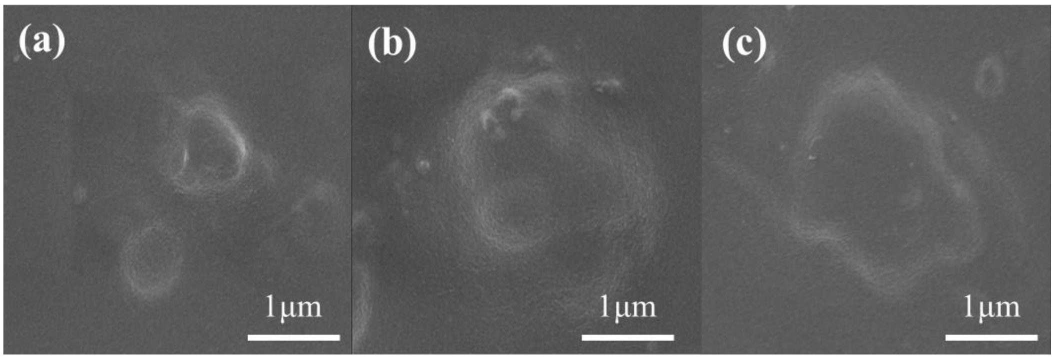

Figure S2. SEM surface image of the ZIF-8@BNNS/Pebax membrane loaded with (a) 5 wt% (b) 10 wt% (c) 15 wt%

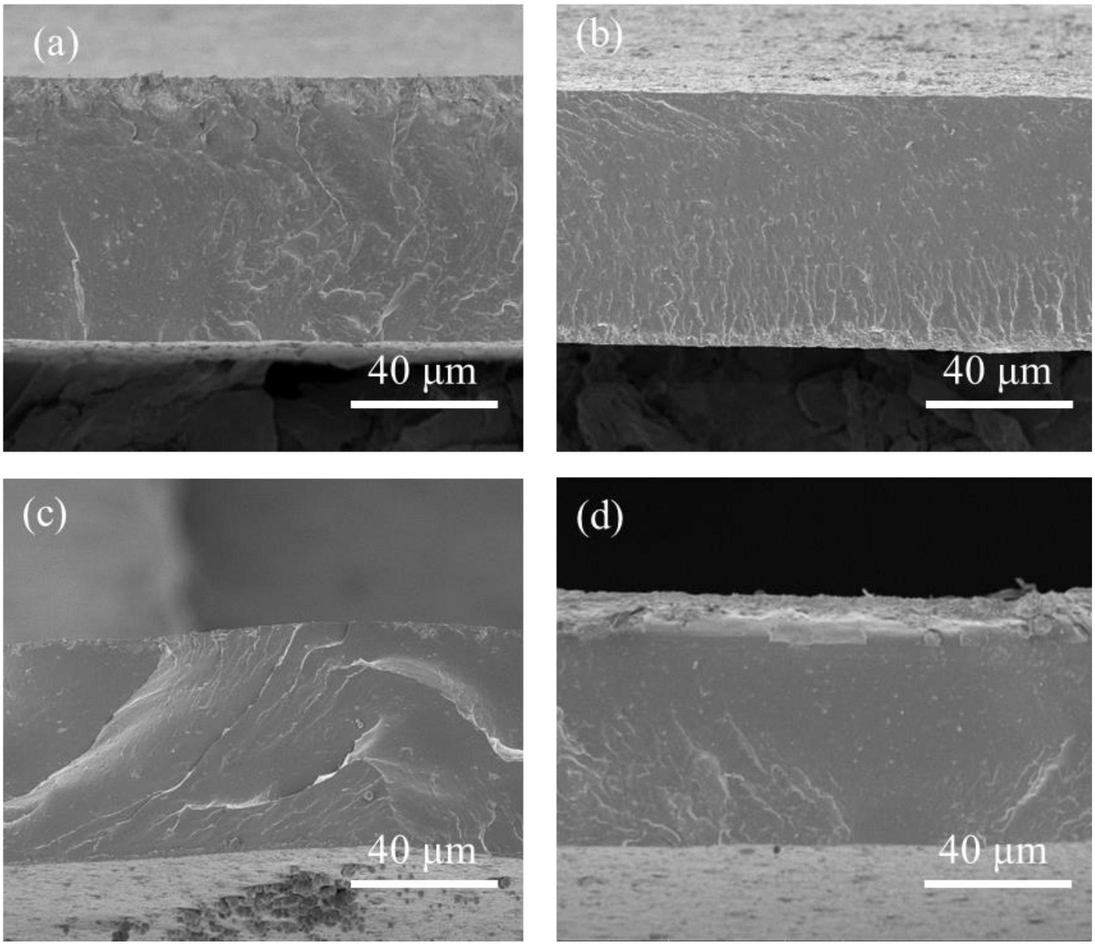

Figure S3. SEM cross-section image of the ZIF-8@BNNS/Pebax membrane loaded with (a) 5 wt% (b) 10 wt% (c) 15 wt% (d) 20 wt%

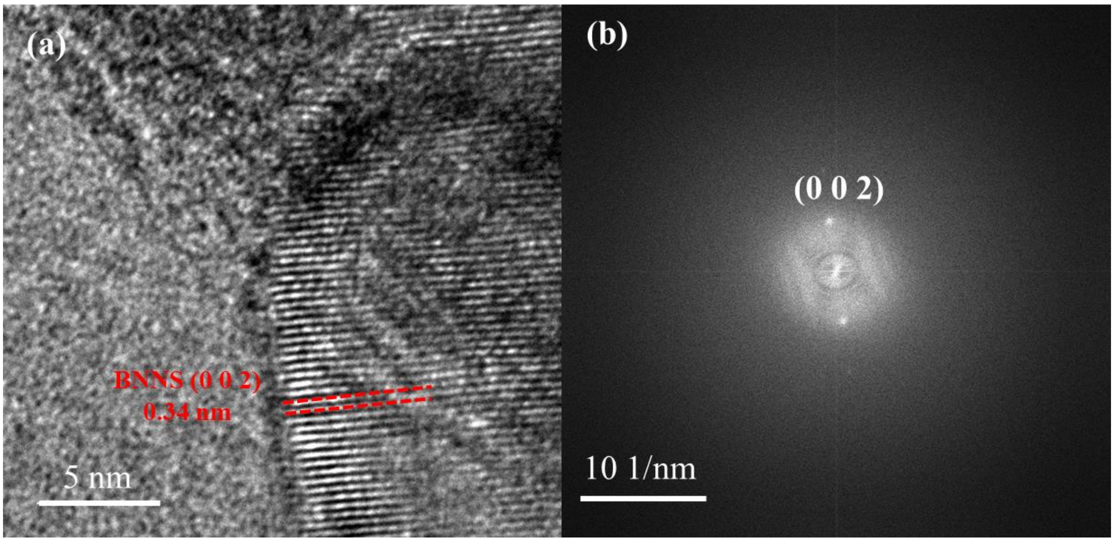

Figure S4. HRTEM images (a) of the ZIF-8@BNNS corresponding SAED pattern (b)

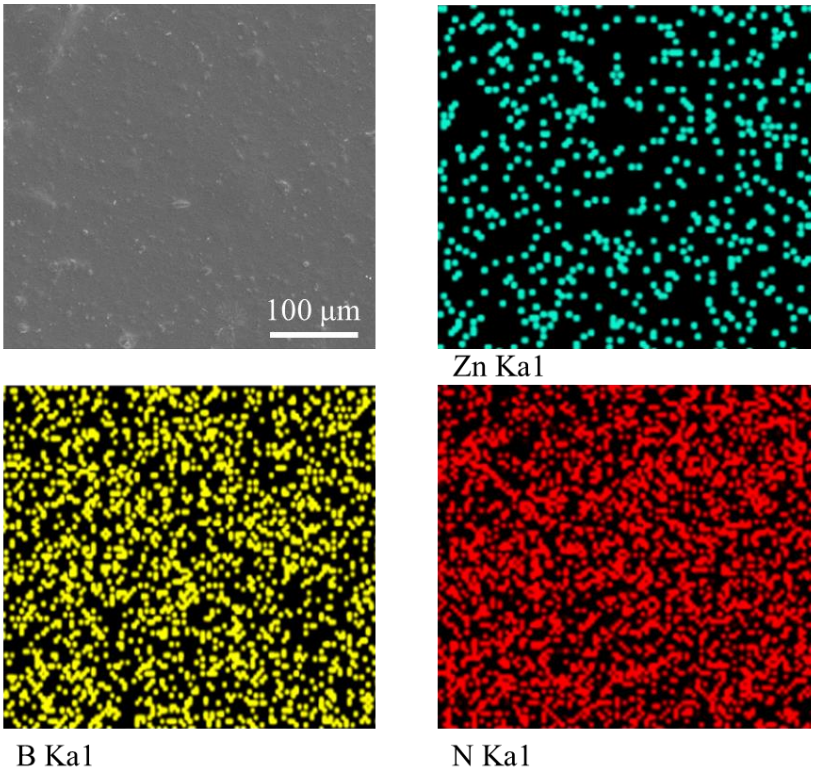

Figure S5. SEM image and EDS mapping (Zn and B) of MMM loaded with 20 wt%

Table S1. CO_2 permeability and CO_2/N_2 selectivity of MMMs with different BNNS loadings and different pressures

**Loadings (wt%)**		**Pressure (bar)**		
		1	3	5
0	P_{CO2}	78.73	80.97	81.97
	$S_{CO2/N2}$	47.98	48.37	48.79
0.5	P_{CO2}	60.91	62.89	63.42
	$S_{CO2/N2}$	54.38	55.91	56.21
1	P_{CO2}	43.98	45.03	45.96
	$S_{CO2/N2}$	62.86	63.56	64.01
2	P_{CO2}	37.65	38.86	39.62
	$S_{CO2/N2}$	56.96	57.62	58.17
